# Terbinafine Resistance in Dermatophytes: A French Multicenter Prospective Study

**DOI:** 10.3390/jof8030220

**Published:** 2022-02-23

**Authors:** Alicia Moreno-Sabater, Anne-Cécile Normand, Anne-Laure Bidaud, Geneviève Cremer, Françoise Foulet, Sophie Brun, Christine Bonnal, Nawel Aït-Ammar, Arnaud Jabet, Aymen Ayachi, Renaud Piarroux, Françoise Botterel, Sandrine Houzé, Guillaume Desoubeaux, Christophe Hennequin, Eric Dannaoui

**Affiliations:** 1 Service de Parasitologie-Mycologie, Hôpital Saint-Antoine, AP-HP, 75012 Paris, France; arnaud.jabet@aphp.fr (A.J.); christophe.hennequin-sat@aphp.fr (C.H.); 2 Centre d’Immunologie et des Maladies Infectieuses, (CIMI-PARIS), Inserm U1135, Sorbonne Université, 75013 Paris, France; 3 Service de Parasitologie-Mycologie, Hôpital La Pitié-Salpêtrière, AP-HP, 75013 Paris, France; annececile.normand@aphp.fr (A.-C.N.); renaud.piarroux@aphp.fr (R.P.); 4 Unité de Parasitologie-Mycologie, Service de Microbiologie, Hôpital Européen Georges-Pompidou, AP-HP, 75015 Paris, France; anne-laure.bidaud@aphp.fr; 5 Laboratoire Bioclinic, 75009 Paris, France; genevieve.cremer@bioclinic.fr; 6 Service de Parasitologie-Mycologie, Hôpitaux Universitaires Henri Mondor, AP-HP, 94000 Créteil, France; francoise.foulet@aphp.fr (F.F.); nawel.ait-ammar@aphp.fr (N.A.-A.); francoise.botterel@aphp.fr (F.B.); 7 UR Dynamic 7380, UPEC, EnvA, USC ANSES, Faculté de Santé, 94000 Créteil, France; 8 Service de Parasitologie-Mycologie, Hôpital Avicenne, AP-HP, 93009 Bobigny, France; sophie.brun@aphp.fr; 9 Faculté de Médecine, Université Sorbonne Paris Nord, 93009 Bobigny, France; 10 Service de Parasitologie-Mycologie, Hôpital Bichat-Claude Bernard, AP-HP, 75018 Paris, France; christine.bonnal@aphp.fr (C.B.); aymen.ayachi@aphp.fr (A.A.); sandrine.houze@aphp.fr (S.H.); 11 Institut Pierre Louis d’Épidémiologie et de Santé Publique, Inserm, Sorbonne Université, 75013 Paris, France; 12 MERIT IRD 261, Université de Paris, 75006 Paris, France; 13 Parasitologie-Mycologie-Médecine Tropicale, CHRU de Tours, 37000 Tours, France; guillaume.desoubeaux@univ-tours.fr; 14 Centre de Recherche Saint-Antoine, CRSA, Inserm, Sorbonne Université, 75012 Paris, France; 15 Faculté de Médecine, Université de Paris, 75006 Paris, France

**Keywords:** terbinafine, resistance, dermatophytes, *Trichophyton*, *Trichophyton indotineae*

## Abstract

In recent years, we have moved from the sporadic description of terbinafine-resistant (TerR) *Trichophyton* spp. isolates to the Indian outbreak due to *T. indotineae*. Population flows have spread TerR worldwide, altering local epidemiology. We conducted a prospective multicentric study to determine the relative frequency of TerR isolates in France (Paris area) and of the newly introduced *T. indotineae* species. TerR isolates were screened by the terbinafine-containing-agar-medium (TCAM) method and confirmed by EUCAST. Sequencing methods were used to identify isolates to the species/genotype level and to analyze substitutions in the squalene epoxidase gene (*SQLE*). In total, 3 isolates out of 580 (*T. rubrum* *n* = 1; *T. interdigitale* *n* = 1; *T. indotineae* *n* = 1) grew on TCAM, showed terbinafine resistance by EUCAST and harbored the Phe397Leu (*n* = 2) or Leu393Ser (*n* = 1) substitution in the *SQLE*. ITS-sequencing of isolates of the *T. mentagrophytes*/*interdigitale* complex (*n* = 125) revealed a relative frequency of 4.8% for *T. indotineae* and the presence of *T. mentagrophytes* genotype VII. Despite the detection of terbinafine resistance, isolates from this complex remained susceptible to itraconazole, voriconazole and amorolfine. Terbinafine resistance is present in France and the dermatophyte epidemiology is changing. Efficient systems must be implemented to survey the evolution of newly introduced species and to identify TerR isolates.

## 1. Introduction

The main etiological agents of dermatophytosis of skin and nails in humans are *Trichophyton rubrum*, *T. interdigitale* and *T. mentagrophytes* [1]. Terbinafine is the first-line treatment for these superficial mycoses. In recent years, we have moved from sporadic cases of terbinafine-resistant (TerR) *Trichophyton* spp. isolates [2,3,4,5,6,7,8,9,10] to an Indian outbreak of terbinafine-resistant tinea corporis and tinea cruris [11,12,13]. This new clinical entity is thought to be a consequence of the irrational use of over-the-counter corticosteroid–antifungal combinations, resulting in the emergence of TerR isolates. The molecular basis for this TerR phenotype has been associated with different point mutations in the gene encoding the squalene epoxidase enzyme (*SQLE*), which plays a role in the ergosterol biosynthesis pathway [14]. Single amino acid substitutions of the *SQLE* protein, such as Leu393Phe or Phe397Leu, are the most commonly identified substitutions [15].

The exact identification of the species responsible for the Indian outbreak, *T. interdigitale* or *T. mentagrophytes*, is controversial [16,17]. In fact, molecular analysis of the responsible strains suggests that we are probably dealing with a new species called *T. indotineae* [18]. Although *T. indotineae* accounts for the majority of cases, cases of TerR *T. rubrum* have also been reported in the Indian outbreak [19]. Afterwards, population flows, associated with increased travel and migration, have allowed the spread of TerR isolates to different European countries [20,21,22] altering the local epidemiology of dermatophytes [23]. We and others have recently reported a series of TerR dermatophytoses due to *T. indotineae* in France [24,25] but the current relative frequency of TerR and of *T. indotineae* isolates remains unknown. Therefore, we conducted a prospective multicenter study to (i) determine the relative frequency in the Paris area, in France, of TerR clinical isolates implicated in onychomycosis, tinea pedis, cruris and corporis, (ii) gain insights into the current epidemiology of *T. interdigitale*, *T. mentagrophytes*, and *T. indotineae* and (iii) determine their susceptibility to four antifungal agents (terbinafine, itraconazole, amorolfine and voriconazole).

## 2. Materials and Methods

### 2.1. Participating Centers and Dermatophyte Isolates

Between January and September 2021, seven diagnostic laboratories from Paris area, (six from university hospitals and one from a private laboratory) participated in this study. Clinical isolates were obtained prospectively from patients with superficial mycosis: onychomycosis, tinea pedis, tinea manus, tinea cruris or tinea corporis dues to *T. rubrum*, *T. interdigitale* and *T. mentagrophytes*. Species identification was initially determined by considering macroscopic and microscopic characteristics of fungal colonies and clinical localization [26]. Using this methodology, only *T. rubrum*, *T. interdigitale* and *T. mentagrophytes* were retained. All isolates from the *T. mentagrophytes*/*interdigitale* complex were kept and stored using 10% of dimethyl sulfoxide (DMSO) in sabouraud liquid medium (VWR) medium at −80 °C until further analysis.

Patient information regarding lesion localization and travel were collected and analyzed when available. The authors testify that all procedures contributing to this work followed the ethical standards of the Helsinki Declaration of 1975, as revised in 2008. Since the study was conducted on isolates collected through routine clinical work and patient’s identifiable information had already been anonymized, no written or verbal informed consent was necessary for patients to participate in this study.

### 2.2. Antifungal Susceptibility Testing

Initial screening for TerR clinical isolates was performed by the participating centers using a terbinafine-containing agar medium method (TCAM) as previously described [14]. For all clinical isolates, fungal growth was tested on sabouraud dextrose agar (SDA) plate containing 0.2 μg/mL of terbinafine and on a drug-free control SDA plate. Visual examination of fungal growth was performed after 7 and 14 days.

Minimum inhibitory concentration (MIC) of terbinafine, itraconazole, amorolfine and voriconazole (all reagents from Sigma-Aldrich) were determined using the European Committee for Antimicrobial Susceptibility Testing (EUCAST) microdilution broth method for microconidia-forming dermatophytes [27,28]. Isolates preliminary screened as TerR as well as all the isolates from the *T. mentagrophytes*/*interdigitale* complex (whatever their susceptibility profile by the screening) were tested by this method. For inoculum preparation, isolates were subcultured on SDA supplemented with cycloheximide and chloramphenicol and incubated at 25 °C for 7 days. For the TerR *T. rubrum* screened isolate, subculture on potato-dextrose-agar medium incubated under 20% CO_2_ was used to induce sporulation [29]. Drug concentrations tested ranged from 0.016 to 8 µg/mL and microtiter plates were incubated at 27 °C for 5 days. The MIC was determined spectrophotometrically with a 50% growth inhibition endpoint. *T. indotineae* SSI-9363 was used as quality control strain. Isolates were defined as resistant when MIC values were above the ECOFFs (https://www.eucast.org/astoffungi/clinicalbreakpointsforantifungals/, accessed on 5 January 2022).

### 2.3. Molecular Identification of Isolates from the T. mentagrophytes/interdigitale Complex

DNA from isolates of the *T. mentagrophyte*/*interdigitale* complex was extracted using an eMAG^®^ (BioMérieux, Capronne, France), after an incubation of a portion of the isolate for a minimum of 10 min in the Lysis buffer (BioMérieux, France). The identification of isolates from the *T. mentagrophyte*/*interdigitale* complex was confirmed down to the species and genotype level by PCR sequencing the ribosomal DNA (rDNA) internal transcribed spacer (ITS). ITS sequencing was performed using the ITS1 (5′-TCCGTAGGTGAACCTGCGG-3′) and ITS4c (5′-TCCTCCGCTTATTGATATGC-3′) couple of primers. PCR amplification was performed using the ready to use mix LC480 (Roche, Diagnostics, Meylan, France), with 0.3 µL of primers at 10 pmol/µL, in a 30 µL mix, and 2 µL of DNA extract. The amplification program consisted on an initial denaturation step of 10 min at 94 °C, 40 cycles of 20 s of denaturation at 94 °C, 30 s of annealing at 55 °C, and 60 s of elongation at 72 °C with a final elongation step of 7 min at 72 °C. The obtained sequences were edited and BLASTed against GenBank database (https://blast.ncbi.nlm.nih.gov/Blast.cgi/, accessed on 20 November 2021) using standard criteria for a significant match. All obtained sequences were deposited in GenBank under the accession numbers OK632108 to OK632231. Genotyping of the isolates was performed by MUSCLE alignment of the ITS sequences using Mega X program (version 10.0.5) with reference sequences previously published [26]. A phylogenetic tree was constructed using Mega X program and iTOL web application (https://itol.embl.de/, accessed on 20 November 2021).

### 2.4. SQLE Sequencing

The *SQLE* gene of the 3 TerR and 20 other selected clinical isolates (all *T. mentagrophytes* and *T. indotineae*; 2 *T. rubrum* and 10 *T. interdigitale* randomly selected among the susceptible isolates) was amplified as previously described [14]. As the amplified fragment is more than 1000 nucleotides long, it was cut in two for sequencing, and a total of four primers were used for the sequencing PCR: Tr*SQLE*-F1, Tr*SQLE*-R1, erg1_2-F and erg1_2-R [25]. The amplification program consisted of an initial denaturation step of 10 min at 94 °C, 40 cycles of 20 s of denaturation at 94 °C, 30 s of annealing at 60 °C, and 60 s of elongation at 72 °C with a final elongation step of 7 min at 72 °C. Sequences were aligned using the sequence GenBank MT700509.1 (*T. mentagrophytes* isolate 203513/19) and the sequence GenBank XM_003233797.1 (*T. rubrum* CBS 118892). Missense mutations were screened using MEGA X (version 10.0.5). *SQLE* sequences were deposited in GenBank under the accession numbers OL415202 to OL415222.

## 3. Results

### 3.1. Terbinafine Resistance Relative Frequency

Between January and September 2021, a total of 580 clinical isolates were included in this study (Table 1). According to the morphological (macro- and microscopic) characteristics of the colonies and the localization of clinical lesions, 436 isolates were identified as *T. rubrum* (75.17%) and 144 belonged to the *T. mentagrophytes*/*interdigitale* complex (24.82%).

Initial screening of the 580 isolates for terbinafine resistance was performed using the TCAM method. A total of 3 isolates out of 580 (0.52%) were able to grow on the terbinafine containing plates. One resistant isolate was initially identified as *T. rubrum* (*n* = 1) using morphological characteristics leading to a relative frequency of resistance of 0.23% (1/436). Two other resistant isolates were identified as *T. interdigitale* (*n* = 1), and *T. mentagrophytes* (*n* = 1) according to morphological characteristics and clinical localization (Table 2). Molecular analysis revealed that resistant *T. interdigitale* isolate belonged to the genotype I (*T. interdigitale*-I) and the resistant *T. mentagrophytes* isolate was indeed *T. indotineae*.

Patients infected with TerR isolates of *T. indotineae* and *T. rubrum* had both returned from a trip to India, whereas the patient infected with the TerR *T. interdigitale*-I had not traveled outside Metropolitan France. For these three isolates, antifungal susceptibility testing using the EUCAST method showed terbinafine MIC higher than 2 μg/mL, but low MIC for itraconazole, voriconazole and amorolfine (Table 2). Two patients were successfully treated with itraconazole 200 mg/d as maintenance therapy for 1 to 3 months, and one patient with urea 40% and bifonazole.

### 3.2. T. indotineae, T. interdigitale and T. mentagrophytes Epidemiology, Clinical Features and Risk Factors

**Figure 1:**
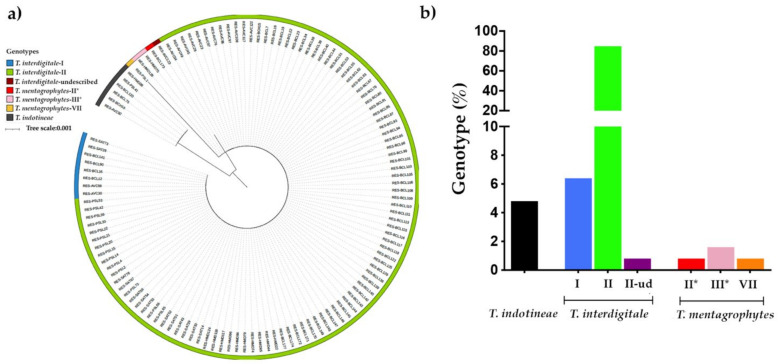
Molecular identification of 125 *T. indotineae*, *T. interdigitale*, and *T. mentagrophytes* isolates collected in Paris area, France. (**a**) Phylogenetic maximum likelihood tree based on ITS-sequences of the clinical isolates from the *T. mentagrophytes*/*interdigitale* complex included in this study. (**b**) Relative frequency of each dermatophyte species and genotype from the *T. mentagrophytes*/*interdigitale* complex. Ud = undescribed.

Of the 144 isolates morphologically identified as belonging to this complex included in this study, 125 were stored and identified by molecular methods. In total, 115 isolates were identified as *T. interdigitale*, 4 isolates as *T. mentagrophytes* and 6 isolates belonged to the recently described species *T. indotineae* (Figure 1).

Phylogenetic analysis of genotypes from *T. interdigitale* and *T. mentagrophytes* isolates showed that the European *T. interdigitale* genotype II was mainly represented (*n* = 106), followed by the genotype I (*n* = 8). We also found a non-previously described genotype related to the genotype II with one-point substitution, A570G, in the ITS region. Concerning *T. mentagrophytes*, the European *T. mentagrophytes* genotype III* (*n* = 2) and genotype II* (*n* = 1) were also detected (Figure 1a,b). A non-previously reported in France genotype, *T. mentagrophytes* genotype VII (*n* = 1), was also identified.

In this study, *T. indotineae* clinical lesions were highly inflammatory and associated with tinea cruris and corporis affecting important body areas. In two patients, *T indotineae* was also associated with hand onychomycosis (*n* = 1) and tinea pedis (*n* = 1). Non- to low-inflammatory tinea pedis or onychomycosis were associated with *T. interdigitale*-I and II. *T. mentagrophytes*-II*, -III* and VII infections were associated with moderately to highly inflammatory lesions of tinea corporis. In patients infected with the *T. mentagrophytes*-VII, highly inflammatory tinea corporis and tinea capitis lesions were observed.

Three of six patients infected with *T. indotineae* had traveled to India, but for the other three patients the clinical skin lesions appeared without leaving France, suggesting a local transmission of this new dermatophyte species. Patients infected with *T. mentagrophytes* VII reported sexual intercourse as a risk factor, and local transmission was also suspected as there was no notion of foreign travel.

### 3.3. Antifungal Susceptibility Testing of T. indotineae, T. interdigitale and T. mentagrophytes Clinical Isolates

The EUCAST method for microconidia-forming dermatophytes was used to determine the antifungal susceptibility to terbinafine, itraconazole, voriconazole and amorolfine of 104 isolates (94 *T. interdigitale*, 4 *T. mentagrophytes*, and 6 *T. indotineae*). As described above (Table 2), two isolates showed terbinafine MIC higher than 2 μg/mL, corresponding to a relative frequency of 1.1% (1/94) and 16.7% (1/6) for *T. interdigitale* and *T. indotineae* species. Despite the detection of TerR isolates, MIC_50_ of the four antifungal agents for the studied population remained low (Table 3). The comparison of results obtained using the EUCAST for terbinafine and the TCAM was consistent. All the isolates identified as susceptible using the latter method had terbinafine MICs lower than 0.125 μg/mL.

**Figure 2:**
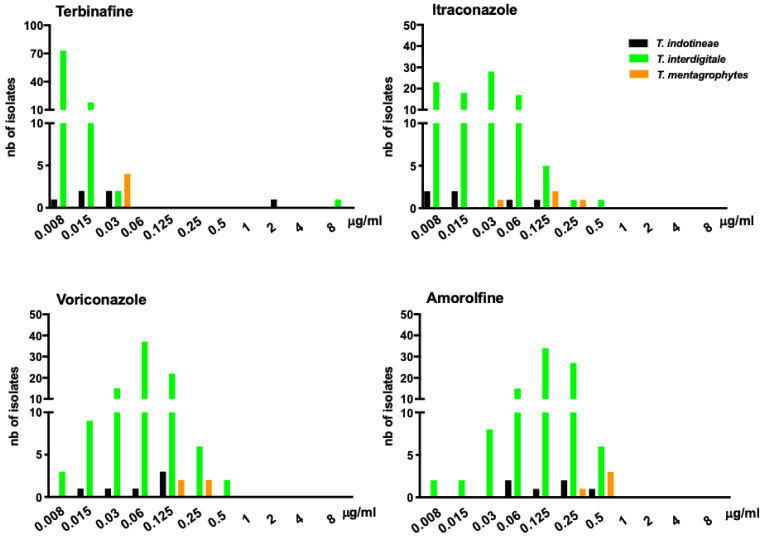
Distribution of terbinafine, itraconazole, voriconazole and amorolfine MIC values for 104 clinical isolates of *T. indotineae*, *T. interdigitale* and *T. mentagrophytes* collected in the Paris area, France. Terbinafine-resistant strains are limited to *T. indotineae* and *T. interdigitale*. MIC distribution shows that isolate population studied remains susceptible to the four antifungal drugs, despite the detection of two TerR isolates. *T. indotineae* *n* = 6; *T. interdigitale* *n* = 94; *T. mentagrophytes* *n* = 4.

MIC distribution was also analyzed for the four antifungal drugs (Figure 2). Terbinafine MIC distribution for *T. interdigitale* showed a higher susceptibility as compared with *T. indotineae* and *T. mentagrophytes*. Concerning itraconazole and voriconazole, all the isolates were wildtype based on ECCOFs to these antifungal drugs, but MICs observed for *T. interdigitale* and *T. indotineae* were lower than those observed for *T. mentagrophytes* (Figure 2). Higher MICs were observed for amorolfine, with *T. mentagrophytes* being the dermatophyte species least susceptible to this antifungal drug (MIC range = 0.25–0.5 μg/mL).

We also studied the correlation between MIC values of terbinafine, itraconazole, voriconazole and amorolfine (Supplementary Figure S1). Our results show a significant correlation between MIC of azoles (itraconazole or voriconazole) and amorolfine (*p* < 0.0001) which was not observed when MIC values of terbinafine were compared with MIC of itraconazole, voriconazole and amorolfine, knowing that a part of the terbinafine MIC distribution was off-scale due to low MICs.

### 3.4. Squalene Epoxidase Gene Mutations

Sequencing of the *SQLE* from the three TerR and 20 susceptible isolates, (all *T. mentagrophytes* and *T. indotineae*; 2 *T. rubrum* and 10 *T. interdigitale* randomly selected among the susceptible isolates) was also performed (Table 4). Missense mutations leading to substituted amino acids in the *SQLE* protein were documented in nine isolates. The TerR *T. indotineae* isolate harbored a Leu393Ser amino acid substitution, whereas *T. indotineae* terbinafine susceptible isolates were wildtype or harbored the Ala448Thr amino acid substitution. No significant differences were observed between MICs for Ala448Thr isolates compared with MICs for wildtype isolates for itraconazole and voriconazole (0.05 μg/mL and 0.09 μg/mL versus 0.04 μg/mL and 0.08 μg/mL, respectively).

TerR *T. rubrum* and *T. interdigitale*-I isolates harbored the Phe397Leu amino acid substitution. Wildtype sequences of the *SQLE* gene were observed in 12 terbinafine susceptible isolates, (*T. rubrum* (*n* = 2), *T. interdigitale*-I (*n* = 5), *T. interdigitale*-II (*n* = 4), and *T. mentagrophytes*-II* (*n* = 1)). In contrast, the Lys276Asp substitution, alone or combined with the Leu419Phe substitution, was observed in *T. mentagrophytes*-III* (*n* = 2) and *T. mentagrophytes*-VII (*n* = 1). All of these isolates had a susceptible phenotype for the four antifungal drugs studied.

## 4. Discussion

Since the onset of the TerR dermatophytosis outbreak, considerable efforts have been made in India and neighboring countries to assess the spread and relative frequency of terbinafine resistance [7,15,19,30,31,32,33]. In Europe, case series of TerR infections mainly due to *T. indotineae* have been recently reported but the real relative frequency has not yet been evaluated. In this multicentric prospective study, terbinafine resistance was observed in one *T. indotineae* isolate but also in *T. rubrum* and *T. interdigitale* isolates, confirming that terbinafine resistance is present in France with a total relative frequency of 0.5% (3/580). When we analyzed our results according to the species, terbinafine resistance relative frequency was 0.23%, 1.1%, and 16.7%, for *T. rubrum*, *T. interdigitale* and *T. indotineae* isolates, respectively, but these percentages should be confirmed with a larger number of *T. indotineae* isolates. In comparison with our previous case series report [25] in which TerR isolates were associated with recalcitrant tinea corporis dermatophytosis due to *T. indotineae*, we have shown in the present study that terbinafine resistance can also be associated with onychomycosis due to *T. interdigitale* or tinea pedis due to *T. rubrum* in the Paris area.

Two cases due to TerR isolates (*T. indotineae* and the *T. rubrum*) were considered as imported and could be associated with the Indian outbreak, whereas the case due to TerR *T. interdigitale*-I was considered as local. Interestingly, local cases of TerR *T. rubrum* harboring Leu397Phe substitution but susceptible to itraconazole, voriconazole and amorolfine have been also recently detected in other French localities (Desoubeaux and Quilliet, personal communication). The selection of TerR isolates locally may be a concern. In France, unlike in India, a medical prescription is required to treat superficial fungal infections with terbinafine, which likely limits the misuse of this antifungal associated with the Indian outbreak. However, the treatment of recurrent onychomycosis, needing repeated course of terbinafine could be a risk factor for selecting resistant isolates as previously described [8,34]. In addition, as griseofulvin is no more commercialized, terbinafine is now the first line treatment of tinea capitis. Therefore, we expect a higher drug selection pressure for terbinafine in the coming years, which implies a high risk of selection of TerR strains worldwide.

The detection of imported cases in different European countries led us to hypothesize that local dermatophyte epidemiology is changing. The inability of classical methods to correctly identify *T. indotineae* together with description of isolates from this species imported into Europe [25] highlights the need to use molecular methods to correctly identify *T. indotineae* species and the different genotypes within the *T. mentagrophytes*/*interdigitale* complex. [18,35]. Our study reveals the presence of European genotypes such as *T. interdigitale*-I, II and *T. mentagrophytes* III* and II*. However, we also detected a high relative frequency of the new species *T. indotineae* (4.8%) and we reported for the first time the presence in France of the genotype *T. mentagrophytes*-VII initially described in Thailand [26] and recently described in Europe [23]. Interestingly, in three of the six infections due to *T. indotineae* cases as well as for the *T. mentagrophytes*-VII case, patients reported that their skin lesions appeared while they were in France and were not related to travel, which reinforces our previous alert that there is local transmission of this newly described dermatophyte species [25]. Overall, these results suggest that the epidemiology of dermatophytes is changing and that *T. indotineae* may become endemic. Compared with a previous study carried out in Europe [23], the percentage of *T. mentagrophytes* was lower in our study and probably related to the fact that clinical isolates were mainly obtained from onychomycoses.

Despite the detection of three TerR isolates, terbinafine, itraconazole, voriconazole and amorolfine MIC_50_ observed in isolates included in this study remained low. TerR isolates showed low MIC for azoles, and two patients were successfully treated with itraconazole. Although voriconazole treatment for TerR cases have been reported [36], the use of this antifungal drugs in invasive fungal infections suggest that this treatment must only be proposed in confirmed TerR cases [37]. Concerning the amino acid substitutions in the *SQLE*, the Phe397Leu and Leu393Ser were detected in our TerR isolates. Other previously described amino acid substitution, such as Ala448Thr [15], Leu419Phe and Lys276Asn [38], were also observed in *T. indotineae* or *T. mentagrophytes* isolates from this study, presenting a susceptible profile to the four antifungal drugs, suggesting that they did not play an important role in terbinafine resistance.

The rapid spread of terbinafine resistance in Europe highlights the importance of antifungal susceptibility testing for dermatophytes [39]. In this study, the TCAM was easily implemented, allowing us to screen 580 isolates from seven differents centers in 7 months. We observed a good correlation between the results obtained with this method and the EUCAST reference method, which confirms its reliability. However, TCAM is a qualitative method that does not accurately determine susceptibility to antifungals. Therefore, in epidemiological studies, the use of the EUCAST method to quantify the susceptibility to antifungal drugs in a representative sample of the studied population could overcome this limitation. Otherwise, the TCAM could be widely implemented in laboratories to allow an easy and cheap detection of TerR isolates. Finally, our study also revealed that dermatophyte identification between closely related dermatophytic species by conventional methods remains challenging and put forward the improvement of available methods, such as PCR or mass spectometry, to allow the correct determination of dermatophyte epidemiology.

In conclusion, terbinafine resistance is present in France, in the Paris area, and dermatophyte epidemiology is changing with the introduction of new species such as *T. indotineae* and new genotypes. Consequently, easy and efficient systems must be implemented to survey the evolution of newly introduced species at a national level and to promptly identify TerR isolates. Dermatologists and mycologists should be alerted to the emergence of TerR dermatophytes and the local transmission of *T. indotineae* in France.

## Figures and Tables

**Table 1:** Frequency and distribution of 580 *Trichophyton* isolates from seven laboratories initially identified according to isolate morphology and clinical localization of lesions.

Identification by Conventional Methods ^1^	Clinical Forms of Dermatophytosis				
	Onychomycosis *n* (%)	Tinea Pedis *n* (%)	Tinea Cruris *n* (%)	Tinea Corporis *n* (%)	Tinea Manus *n* (%)
*T. rubrum* (*n* = 436)	292 (67.0)	112 (25.7)	24 (5.5)	7 (1.6)	1 (0.2)
*T. interdigitale* (*n* = 136)	96 (70.6)	35 (25.7)	0 (0.0)	3 (2.2)	2 (1.5)
*T. mentagrophytes* (*n* = 8)	2 (25.0)	0 (0.0)	1 (12.5)	5 (62.5)	0 (0.0)
Total (*n* = 580)	390 (67.2)	147 (25.4)	25 (4.31)	15 (2.56)	3 (0.52)

^1^ According to morphological characteristics (macroscopic and microscopic identification) and localization of dermatophytosis.

**Table 2:** Antifungal susceptibility and molecular characteristics of three TerR *Trichophyton* isolates and patient information.

Identification		Antifungal Susceptibility MIC (μg/mL)				Patient Information		
Classical Methods ^1^	Molecular Methods ^2^	TRB ^3^	ITR	VRZ	AMO	Patient Origin/Travel	Clinical Form	Treatment
*T. mentagrophytes*	*T. indotineae*	2	0.016	0.06	0.125	India	Tinea corporis	ITR ^4^ 200 mg/d
*T. rubrum*	*T. rubrum*	4	0.063	0.063	0.031	India	Tinea pedis	ITR ^4^ 200 mg/d
*T. interdigitale*	*T. interdigitale-genotype I*	8	0.06	0.125	0.125	France	Onychomycosis	Nail debridement /bifonazole

^1^ According to morphological characteristics (macroscopic and microscopic identification) and localization of dermatophytosis. ^2^ ITS sequencing. ^3^ TRB = Terbinafine; ITR = Itraconazole; VRZ = Voriconazole; AMO = amorolfine. ^4^ ITR maintenance therapy.

**Table 3:** MICs values of 4 antifungal agents for 104 clinical isolates of *T. indotineae*, *T. interdigitale* and *T. mentagrophytes* collected in Paris area, France.

			MIC (μg/mL) ^1^		
		Terbinafine	Itraconazole	Voriconazole	Amorolfine
***T. indotineae* (*n* = 6)**					
	Range	0.008–2	0.008–0.125	0.015–0.125	0.06–0.5
	MIC_50_	ND ^2^	ND	ND	ND
	MIC_90_	ND	ND	ND	ND
	Gmean	ND	ND	ND	ND
***T. interdigitale* (*n* = 94)**					
	Range	0.008–8	0.008–0.5	0.008–0.5	0.008–0.5
	MIC_50_	0.008	0.03	0.06	0.125
	MIC_90_	0.015	0.06	0.125	0.25
	Gmean	0.010	0.025	0.062	0.120
***T. mentagrophytes* (*n* = 4)**					
	Range	0.03	0.03–0.25	0.125–0.25	0.25–0.5
	MIC_50_	ND	ND	ND	ND
	MIC_90_	ND	ND	ND	ND
	Gmean	ND	ND	ND	ND
**Total (*n* = 104)**					
	Range	0.008–8	0.008–0.5	0.008–0.5	0.008–0.5
	CMI_50_	0.008	0.03	0.06	0.125
	CMI_90_	0.015	0.125	0.125	0.25
	Gmean	0.011	0.026	0.064	0.128

^1^ MIC = minimal inhibitory concentration. Gmean = geometric mean. ^2^ MIC_50_, MIC_90_ and Gmean were not determined when the number of isolates per species was <10.

**Table 4:** Squalene epoxidase enzyme sequencing of 3 TerR and 20 susceptible isolates collected in the Paris area, France.

Organism	Genotype	Terbinafine MIC (μg/mL)	Nucleotide Substitution (*SQLE*)	Amino Acid Substitution (*SQLE*)	Accession Number
***T. indotineae***		0.03	Wt	Wt	OL415218
		0.03	G^1342^A	Ala448Thr	OL415219
		0.015	Wt	Wt	OL415223
		0.015	G^1342^A	Ala448Thr	OL415220
		0.008	G^1342^A	Ala448Thr	OL415222
		2	T^1178^C	Leu393Ser	OL415221
***T. rubrum***		ND	Wt	Wt	OL415199
		ND	Wt	Wt	OL415200
		4	C1191A	Phe397Leu	OL415198
***T. interdigitale***	I	0.008	Wt	Wt	OL415202
		0.008	Wt	Wt	OL415203
		0.008	Wt	Wt	OL415206
		0.015	Wt	Wt	OL415207
		0.008	Wt	Wt	OL415211
		8	C1191A	Phe397Leu	OL415213
	II	0.008	Wt	Wt	OL415208
		0.008	Wt	Wt	OK632159
		0.008	Wt	Wt	OL415212
		0.008	Wt	Wt	OL415210
***T. mentagrophytes***	II*	0.03	Wt	Wt	OL415214
	III*	0.03	C^1255^T	Leu419Phe	OL415215
		0.03	C1255T	Leu419Phe	OL415216
			G828C	Lys276Asn	
	VII	0.03	C1255T	Leu419Phe	OL415217
			G828C	Lys276Asn	

ND = EUCAST was not performed for these isolates but both isolates did not grow in the terbinafine containing agar plates.

## Data Availability

The data presented in this study are available on request from the corresponding author.

## Supplementary Materials

The following are available online at https://www.mdpi.com/article/10.3390/jof8030220/s1, Figure S1: Correlation between itraconazole, voriconazole and amorolfine MICs of clinical isolates.
