# Supplementary material for: Terbinafine Resistance in Dermatophytes: A French Multicenter Prospective Study
Source: J Fungi (Basel). 2022 Feb 23;8(3):220. doi: 10.3390/jof8030220 (PMC8948947; doi:10.3390/jof8030220)
Supplement: Supplementary file 1 [file jof-08-00220-s001.zip › jof-1579324-supplementary.pdf]

Journal of
Fungi

Supplemental Figure S1 :

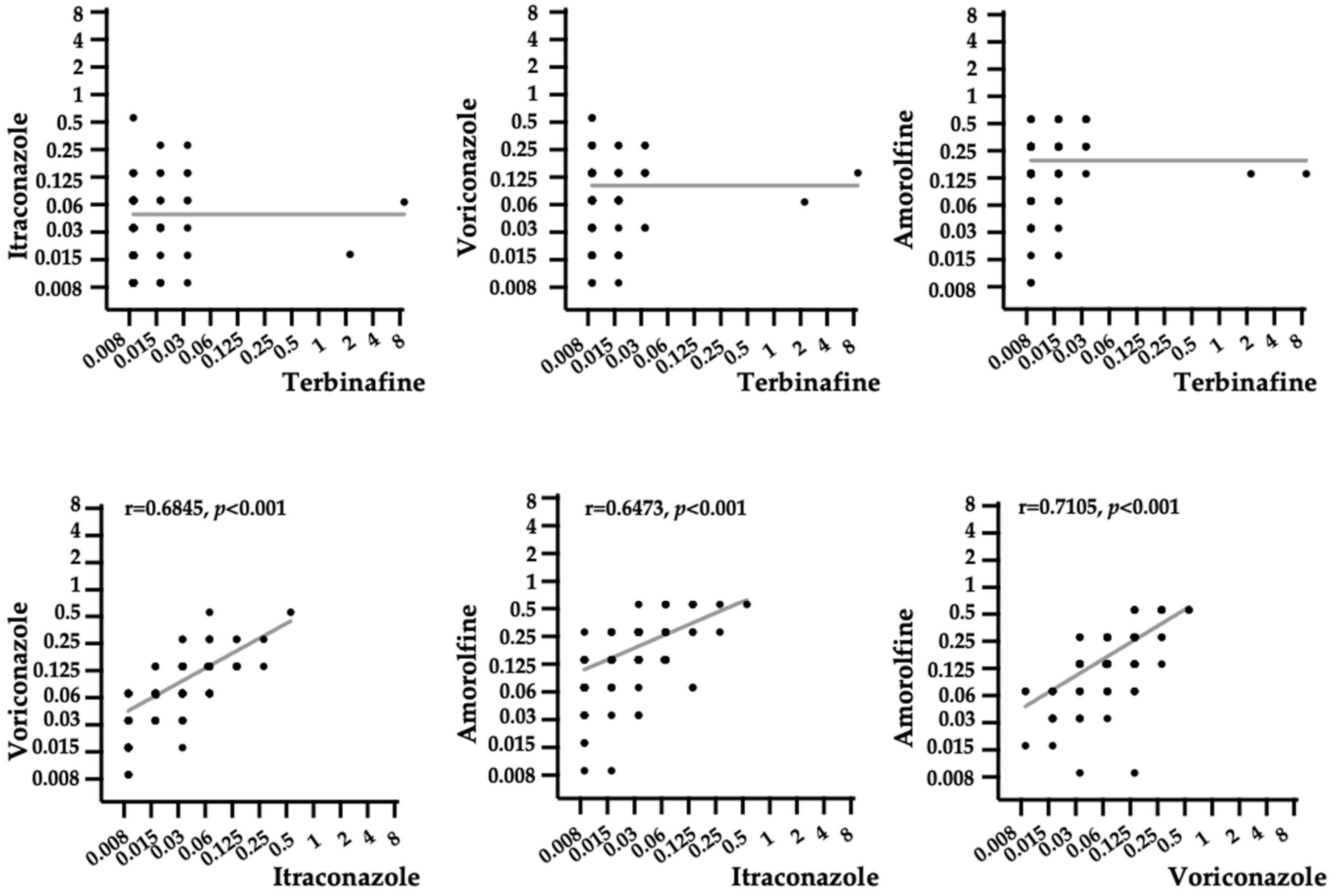

Figure S1. suppl. Correlation between itraconazole, voriconazole and amorolfine MICs of clinical isolates. MICs from each isolate and from each antifungal drug were compared. Grey line shows the simple linear regression between MIC determined using Prism 9 program.
